# Noninvasive Evaluation of Nuclear Morphometry in Breast Lesions Using Multispectral Diffuse Optical Tomography

**DOI:** 10.1371/journal.pone.0045714

**Published:** 2012-09-19

**Authors:** Mohammad Reza Hajihashemi, Stephen R. Grobmyer, Samer Z. Al-Quran, Huabei Jiang

**Affiliations:** 1 Department of Biomedical Engineering, University of Florida, Gainesville, Florida, United States of America; 2 Department of Surgery, University of Florida, Gainesville, Florida, United States of America; 3 Department of Pathology, University of Florida, Gainesville, Florida, United States of America; The Chinese University of Hong Kong, Hong Kong

## Abstract

Breast cancer is the most prevalent cancer and the main cause of cancer-related death in women worldwide. There are limitations associated with the existing clinical tools for breast cancer detection and alternative modalities for early detection and classification of breast cancer are urgently needed. Here we describe an optical imaging technique, called multispectral diffuse optical tomography (DOT), and demonstrate its ability of non-invasively evaluating nuclear morphometry for differentiating benign from malignant lesions. Photon densities along the surface of the breast were measured to allow for the extraction of three statistical parameters including the size, elongation and density of nuclei inside the breast tissue. The results from 14 patients (4 malignant and 10 benign lesions) show that there exist significant contrasts between the diseased and surrounding normal nuclei and that the recovered nuclear morphological parameters agree well the pathological findings. We found that the nuclei of cancer cells were less-spherical compared with those of surrounding normal cells, while the nuclear density or volume fraction provided the highest contrast among the three statistical parameters recovered. This pilot study demonstrates the potential of multispectral DOT as a cellular imaging method for accurate determination of breast cancer.

## Introduction

Alterations in nuclear morphology are a hallmark of cancer cells. Nuclei of cancer cells divide out of control and contain extra chromosomes. Compared with nuclei in non-cancerous cells, nuclei in cancer cells are larger, closer together and more irregular in morphology [1]–[7]. The changes in cellular and nuclear morphology also correlate with tumor grade. Currently, most of the relevant diagnostic techniques for evaluating breast lesiosn are invasive and require biopsy sampling of the breast tissue and subsequent pathologic analysis. The ability to assess changes in nuclear morphology non-invasively would represent a major step forward in the development of strategies to non-invasively evaluate indeterminate breast lesions and detect breast cancer.

Light scattering tools are promising candidates to probe cellular structures. In tissues, light scattering happens when there is a refractive index mismatch between cells or subcellular organelles and their surrounding media. As a result, some portion of the incident light is redirected relative to the scattering particle. Some examples of scattering particles include nuclei, cell membranes, fibers and structures such as the mitochondria [9]–[10]. If the probing wavelength is chosen comparable to dimensions of cellular structures, the resultant optical properties become highly responsive to structural changes [8]. Optical characterization techniques include a broad range of measurement and reconstruction techniques, alternatively called inverse light scattering techniques [8]–[18]. By processing the measured data using these techniques, one can retrieve meaningful information regarding the particles involved [11]. The objective is to minimize the error between some characteristics of the scattered light and those obtained from relevant numerical simulations, thereby retrieval of the morphological parameters of cells and nuclei.

However, successful recovery of cellular morphology is a challenging task because of complex scattering mechanism in tissues and organs. The cells and organelles have irregular shapes, multiple scattering interactions and heterogeneous dielectric properties. The application of light scattering techniques for cell morphologic characterization has been considered in several publications [8]–[9], [12]–[18]. Methods like scattering spectroscopy are more suitable for superficial imaging since they were not accompanied by realistic scattering models and efficient inversion schemes. In Ref. [8], an interesting application was reported using angle-resolved low coherence interferometry to detect dysplasia in Barrett's Esophagus.

Separate studies suggest that two types of organelles mainly contribute to light scattering in the breast tissue. It has been shown that small structures, such as the mitochondria cause the primary large-angle scattering [14]. Another study revealed that the nuclear size distribution could be retrieved from measurements of the light's intensity [15]. However, since both signals appear in the scattered light, this apparent contradiction is justified [9].

To analyze light propagation and scattering in tissue, we adopted diffuse optical tomography (DOT) based on the diffusion of near-infrared light through biological tissue. In DOT, wave phenomena such as interference and polarization are ignored and only the transport of light energy is considered [11]. The advantage of using near-infrared light (600–1000 nm) is that photon propagation is dominated by scattering rather than absorption. Furthermore, it is better suited for characterization of nulcei deeply located in the breast tissue [11]. Generally, measurement data at a single wavelength do not contain enough information to recover nuclear morphology parameters, so our multispectral diffuse optical tomography system collects photon densities from the breast skin at multiple wavelengths.

One of the main limitations of previous works has been that cells and nuclei were modeled as homogenous dielectric spheres and interactions between cellular structures were not taken into account [18]. In a significant step further to utilize a more realistic scattering model, we demonstrate here that more reasonable morphological parameters are obtained upon modeling nuclei as spheroidal particles, randomly oriented in the breast tissue. In this work, improvements are obtained for both the recovered nuclear size and the volume fraction, and the results are compared with the microscopic images. Furthermore, we show that the nuclear aspect ratio provides an additional contrast mechanism for breast cancer detection. In prior studies, using one-particle system, the results had large discrepancies with the pathological images [18]. Therefore, a bi-modal scattering system was employed to consider the scattering from both larger particles such as nuclei and smaller particles such as nucleoli and mitochondria. However, this approach was case-dependent and errors were still large in some cases. In addition, it was difficult to estimate the contribution from each part in the scattering response. In this work, we use a one-particle scattering system with the same reconstruction parameters in all cases. This approach is more suitable for tumor identification and classification purposes. As a result, the nuclear size, volume fraction and the aspect ratio are quantitatively recovered with small reconstruction errors.

In our method, *in vivo* photon density measurements are first used to recover the reduced scattering spectra using a finite element-based reconstruction algorithm [11]. Consequently, the reduced scattering spectra are used to extract nuclear morphology parameters using our recently developed T-matrix-based algorithm [19]–[20]. The parameters to be retrieved include nuclear volume fraction, mean nuclear size and aspect ratio at each node of a circular imaging plane. Cross-validations of our results are performed using microscopic images as well as pathological findings from the literature [1]–[7]. Although the T-matrix method has been previously reported to probe cellular structures, this work represents the first to address application of this method for breast cancer detection and classification via nuclear morphometry deep inside the breast tissue.

In this work, the clinical data, which were already collected and processed by our group, are reanalyzed using a new cellular imaging algorithm. Compared with the work reported in Ref. [18], the proposed algorithm can recover an additional nuclear morphology parameter: the aspect ratio which is the measure of nuclear elongation. Further, the nuclear size and volume fractions are recovered more accurately. In future we plan to test our new algorithm using larger set of clinical data.

## Methodology and Algorithm

### Clinical data collection and the particle characterization algorithm

**Figure 1:**
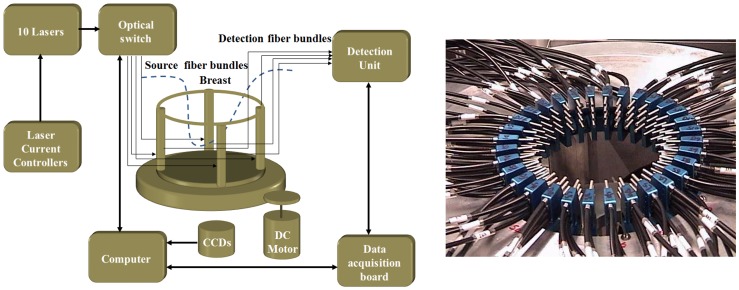
(a) Schematic of the multispectral DOT system. (b). Photograph of the breast/fiber optic array.

The first step in our algorithm involves collecting photon density measurements at the breast skin using our multispectral DOT system shown in Fig. 1. In Fig. 1b, a interface of breast/fiber optic array is shown. The multispectral DOT system, as described in [21], consists of ten continuous-wave laser modules transmitting near infrared light to an optical switch and subsequently to 16 pre-selected points on the breast surface. The photon density measurements at 256 possible source/detector pairs are then imported to DOT reconstruction algorithm [11]. The data are processed at the following eight wavelengths: 638 nm, 673 nm, 690 nm, 733 nm, 775 nm, 808 nm, 840 nm and 915 nm. Using the photon diffusion equation, measured data and a 634-node triangular finite element mesh, the DOT reconstruction algorithm retrieves two-dimensional maps of the reduced scattering coefficient, as well as the absorption coefficient, at the wavelengths of interest [11]. Afterwards, we employ our T-matrix-based inverse algorithm to recover the three statistical parameters characterizing nuclear morphology and its concentration. The T-matrix method is a fast and powerful technique for computing the optical properties of non-spherical particles [22]. Our particle characterization algorithm was elaborated in Ref. [19], and corroborated with tissue-mimicking phantom measurements in Ref. [20].

In summary, the objective is to extract the nuclear size, volume fraction and the aspect ratio using the following expression which relates the bulk reduced scattering coefficient of the breast tissue to the parameters of interests at multiple wavelengths.
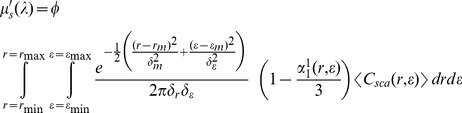
(1)where 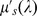
is the reduced scattering coefficient of breast tissue at the wavelength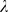. The equal-volume radius of the scattering particle varies in the range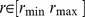. The aspect ratio is represented by the symbol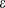 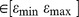. The aspect ratio is the ratio of the equatorial radius to the polar radius of the spheroidal particle. We assume that spheroidal nuclei have random orientations within the breast tissue.

The other assumption is that a bivariate normal distribution function describes the random perturbations in the nuclear size and aspect ratio and there is no statistical correlation between these two parameters. The mean values of the particle radius and aspect ratio are represented by the symbols 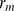
and 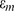, respectively. The concentration of particles is represented by the symbol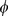, which represents the number of particles per cubic millimeter. The volume fraction is related to the concentration of particles through the following equation:
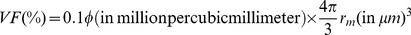
(2)



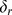
 and 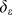
are the standard deviations of the particle size and aspect ratio, respectively. In this work we assumed that the standard deviations equal to 10% of their corresponding mean values.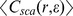 denotes the averaged scattering cross section of an ensemble of randomly-oriented monodisperse spheroidal particles, having an equivalent radius of *r* and aspect ratio of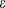. 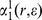 is a coefficient obtained by expanding the phase function [19].

Our inverse algorithm is based on iterative Newton method, accompanied with a regularization scheme, which minimizes the discrepancy between the observed reduced spectra 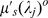
and the calculated ones 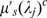 using Eq. 1, where 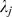 *j = *1, 2 … are the measurement wavelengths.
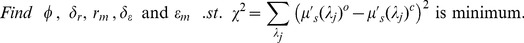
(3)


If the vector 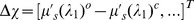 represents the error between the observed and calculated reduced scattering coefficients and 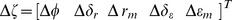
is the vector which updates the parameters of interest, then according to Newton's method, 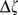
is calculated by solving the following matrix equation:
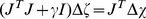
(4)where
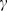
is an appropriately chosen regularization parameter and *I* is the identity matrix [19]. The elements of the Jacobian matrix *J* are determined by direct differentiation of Eq. (1) with respect to the model parameters.

Our algorithm requires a T-matrix database consisting of the values of reduced scattering cross section with different combinations of the nuclear size and aspect ratio. The reduced scattering cross sections are *a priori* computed and stored [22]–[23]. In our database, nuclear size varies in the range of 2 µm–12 µm with the step of 0.1 µm and the aspect ratio varies in the range of 0.5–1.1 with the step of 0.05. The refractive indices of nuclei and the surrounding breast tissue are assumed to be 1.45 and 1.37, respectively [24].

At each node, the objective of inverse algorithm is to minimize the residual sum of squares between simulated and measured reduced scattering coefficient. Since the performances of Newton-based optimization techniques, including ours, are sensitive to initial values chosen for the unknown parameters, we preceded the reconstruction with an initial search algorithm which finds the “best fit” of parameters minimizing an error function [19]. We have used two initializations for the cancer and healthy regions. Since both regions have irregular boundaries, the criteria for assigning the nodes to each region is chosen based on the average value of reduced scattering coefficient.

### The patients under study

In this following section, summary reports of mammography, biopsy and ultrasonography performed for three cancer patients and three benign patients, are provided.

Two patients with malignant breast cancer (ID# G1 and G2) were identified to have invasive ductal carcinoma and the other one (ID# G8) had ductal carcinoma *in situ*. Since ductal carcinoma *in situ* is frequently treated similar to the breast tumor, DCIS cases are considered among malignant types in this work. The patients with benign lesions had nodule, calcification or cyst in their breasts.

### Cancer cases

#### Patient G1

The first patient was a 52 years old female volunteer. For her right breast, we conducted the near-infrared (NIR) diffuse optical tomography. Also craniocaudal (CC), mediolateral oblique (MLO) mammograms and sonographic images were obtained. By observing mammograms, we found an ill-defined speculated mass in lateral portion of her right breast and the BI-RADS score was 4. According to sonographic images, there was an ill-defined hypoechoic mass with lobular margins with the size of ∼ 1.0×1.6×1.0 cm in the 9 o'clock position which agrees with the abnormality noted in the mammogram. Afterwards mastectomy was performed and the surgery indicted that the patient had an invasive ductal carcinoma in the right breast. A firm and retracted nodule of pink-tan tumor tissue, with the size of ∼1.2×1.2×1.3 cm, was revealed in the lower outer quadrant subject to the biopsy site on the skin. The DOT imaging was performed one week earlier than the biopsy and mastectomy surgery. By processing the DOT scattering images, one target was detected around 6 o'clock whereas the surgery report indicated the tumor was in the lower outer quadrant, around 7 o'clock position.

#### Patient G2

The second patient was a 50 years old volunteer with palpable abnormality, again in the right breast. An extremely dense pattern of fibroglandular tissue was discovered from the CC and MLO mammogram views, which limits the sensitivity. We found a 4–5 cm mass-like area in the upper-outer quadrant of the right breast, associated to the palpable abnormality. There was no architectural distortion or suspicious calcification. Moreover, no adenopathy or skin thickening was noted. The ultrasonography of the right breast revealed a 3×1.9 cm heterogeneous hypoechoic mass with some posterior shadowing corresponding to the palpable abnormality located at 10 o'clock. There was an adjacent hypoechoic solid nodule, ∼9×6 mm, and some other small nodules. After biopsy, mastectomy was performed. From the surgery, a white, semi-cystic, semi-solid tumor mass with infiltrative borders was located in the lateral inferior quadrant (6–9 o'clock). It was measured 4.0 cm from lateral to medial, 4.5 cm from inferior to superior, and 2.4 cm form anterior to deep. The breast parenchyma revealed fibrocystic change underneath the areola and nipple, which created a solid tan-white portion of breast tissue, ∼ 2.2×3.0×2.5 cm. The NIR DOT imaging of the second patient was conducted two days before the biopsy and surgery. From the scattering images, we observed that the detected target was not limited in one region. The detected targets were located either in the center or around 10 o'clock of the breast. These findings were consistent with the surgery report.

#### Patient G8

The third patient was a 50 years old female volunteer. The reports of MLO and CC mammograms of both breasts were obtained. The breasts were composed of very dense fibroglandular tissues, limiting the mammogram sensitivity. There were no masses or areas of distortion. The mammograms' conclusion was “Probably benign right mammogram. Benign left mammogram.” However, the biopsy report indicated a ductal carcinoma *in situ*, non-comedo type and the nuclear grade was found to be III. The biopsy found there were 2 masses in the left breast, one in the 11 o'clock position and one in the retroareolar region. Only the 11 o'clock lesion was biopsied and surgical excision was recommended. From both the scattering images and the morphological images, the detected targets were located around 12 o'clock and 3 o'clock. There was more than one target, confirming the biopsy report, while the mammogram report indicated that there was no suspicious mass or lesion in the left breast.

### Benign cases

#### Patient S5

The fourth patient was a 69 years old female volunteer. In the mammogram, a stellate area of architectural distortion and asymmetric density had developed in the superior lateral quadrant. The patient felt a palpable mass at this location. However the ultrasound examination for the right breast indicated that no discrete mass was identified in the area of the known mammographic abnormality. There were some minor shadowing in this area. No biopsy report was available for this patient. In all the absorption coefficient images, the scattering images and morphological images, only one target around 8 o'clock was identified, whereas in the mammogram the abnormality was located around 9 o'clock.

#### Patient S7

The fifth patient was a 42 years old female volunteer. According to the mammogram report, no persisted nodular density was observed and reportedly there was no palpable mass. The ultrasound report indicated that two smooth lobulated solid nodules were presented in the medial aspect of the left breast at 9∶30 o'clock. The more anterior nodule measured ∼5.8×4.8×8.3 mm and the mid left breast nodule measured 6.9×3.6×9.1 mm. The BI-RADS category was 3. In the scattering images, there were two targets located around 6∶00 o'clock and 8 o'clock, while the mammogram could not identify any target and the ultrasound detected two separate nodules positioned around 9∶30 o'clock.

#### Patient S9

The sixth patient was a 60 years old female volunteer. According to the mammogram report, a small nodular opacity was observed in a single projection on the exaggerated craniocaudal view of the right breast. Its diameter was ∼1.4 cm and it was placed within the lateral aspect of the right breast. The BI-RADS category was 2, indicating a benign case. Ultrasound imaging was not performed for patient S9. The NIR DOT imaging experiment was performed two days after the mammogram screening.

As well as these cases, the clinical measurements were performed for 7 more benign patients and one more malignant patient. The reports from mammography, ultrasonography and DOT were similar and they are not detailed here.

### Ethics Statement

Our clinical study was approved and monitored by the University of Florida Gainesville Health Science Center Institutional Review Board (http://irb.ufl.edu). Each enrolled patient signed the consent form.

## Results and Discussion

**Figure 2:**
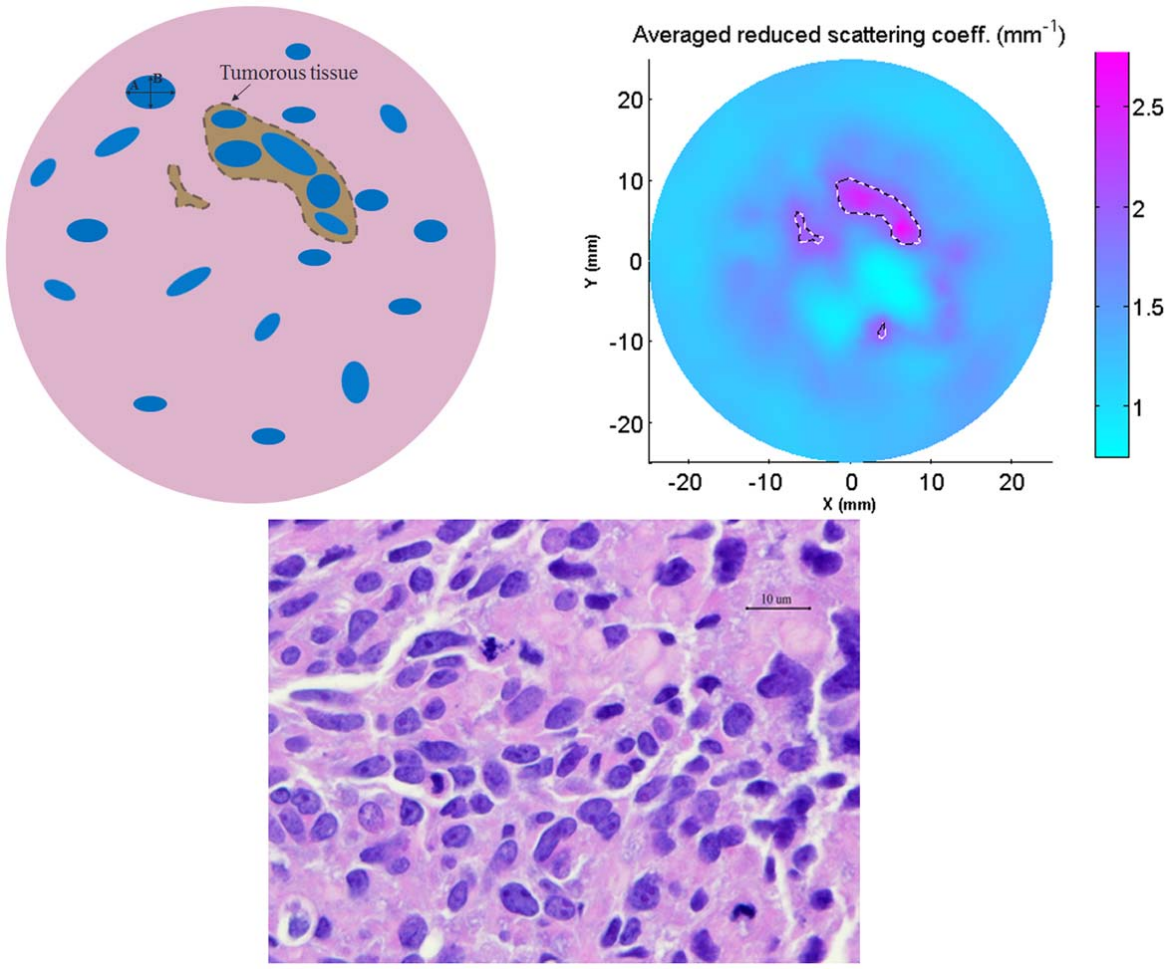
(a). A schematic model for nuclei inside the breast tissue. (b). Averaged reduced scattering coefficient (over all wavelengths). (c) A microscopic image from the tumor region for patient G8. Cancer region and nuclei are circumscribed by dashed lines to aid visualization.

We utilized our technique to recover the nuclear size, volume fraction and aspect ratio of malignant and benign breast lesions, using the same input data of Ref. [18]. More information regarding the demographics of the patients and the diagnoses on which the experiments were performed are given in Ref. [18]. The data measured from three malignant and eleven benign patients are processed and the recovered parameters are averaged out and compared with microscopic images. Nuclei in cancer and healthy regions are modeled as spheroid-shaped particles randomly oriented within the breast tissue as shown in Fig 2a. The polar and equatorial diameters of a spheroid-particle are marked by symbols *A* and *B*, respectively. The nuclear size is defined as 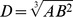 representing the diameter of equal-volume-sphere. The nuclear aspect ratio is defined as *AP = B/A*, which is smaller than one in this work.

We noted that the values of reduced scattering coefficient and the recovered parameters rapidly drop from their maximums at tumor center to their minimums, ∼1 cm away from the tumor boundary, which is considered the healthy tissue.

**Figure 3:**
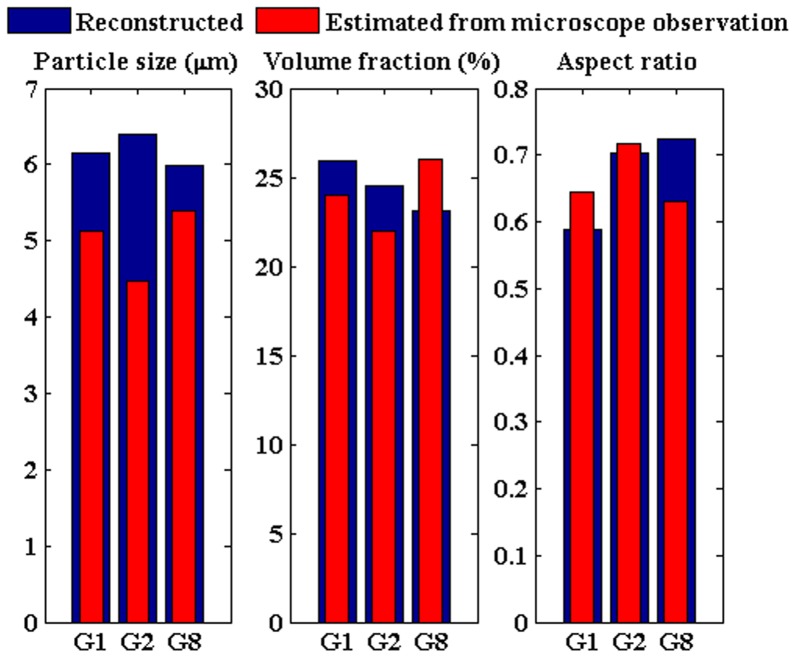
Averaged values of the retrieved parameters along with those estimated from microscope observation of tumor tissue.

**Figure 4:**
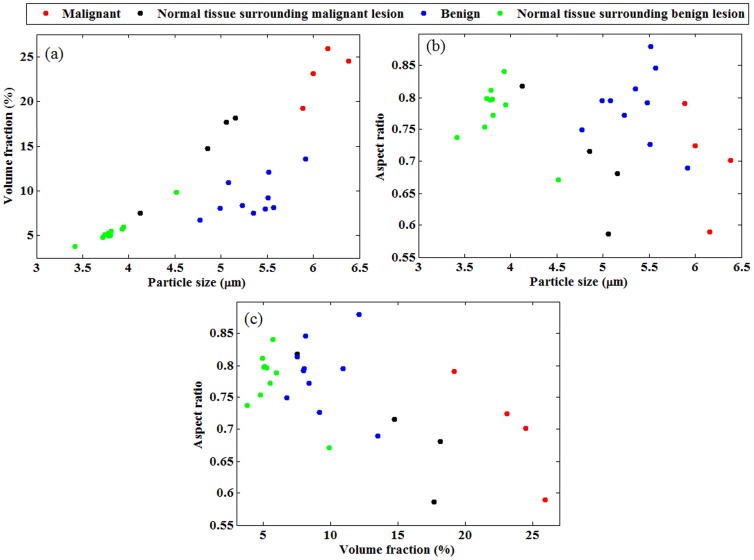
Average values of the three recovered parameters from all patients with malignant or benign lesions and their surrounding normal tissues. (a) mean diameter versus volume fraction. (b) mean diameter versus aspect ratio.(c)volume fraction versus aspect ratio.

Our method can recover 2D maps of the three morphological parameters (nuclear size, volume fraction and the aspect ratio) at all the computational nodes. These nodes may associate with cancer or healthy tissues. The nodes for which values of the averaged reduced scattering coefficient (over all wavelengths) vary in top 40% of the range are assigned to be in cancer tissue and the rest of nodes are assumed to be healthy. These assumptions are in reasonable agreement with the recovered parameters. It should be reminded that the reported numbers in Fig. 3–4 are averaged out at all the nodes in cancer or healthy tissue.

**Table 1:** The nuclear morphology parameters, reported by several researchers.

Reference	Mean particle size (µm)	Volume fraction	Aspect ratio	Tissue type
El Sharkawy et al, [6].	8.4	–	–	Malignant
El Sharkawy et al [6].	5.7	–	–	Healthy
M. Deacu et al [2].	8.6–12.2	–	0.69	Malignant
M. Deacu et al [2].	4.7–6.4	–	0.71	Healthy
Kalhan et al [14].	5.77	–	0.71	Benign
Kalhan et al [14].	6.2–11.2	–	0.68	Malignant
E. Artacho-Pérula [5]	–	20%–30%	–	Malignant
S. Suhane et al [7].	–	10%	–	Healthy
S. Suhane et al [7].	–	20%–40%	–	Malignant, Benign

In Fig. 2b, the average reduced scattering coefficient from patient G8, which was diagnosed with invasive ductal carcinoma, is shown. Since nuclei in cancer regions are larger and more crowded, they scatter the incoming light more strongly. This fact is justified with larger values of the reduced scattering coefficient, shown in Fig. 2b. A microscopic image taken from the tumorous lesion of patient G8 is shown in Fig. 2c. We analyzed Fig. 2c to get the estimates of actual nuclear size, volume fraction and aspect ratio. Counting the pixels lying inside the nuclei region reveals that the nuclear area fraction in the tumorous lesion is ∼26%. Similarly, by analyzing microscopic images from other two malignant cases, the nuclear area fraction is estimated in the range of 22%–26%. The nuclear size is estimated in the range of 4–6 µm and the nuclear aspect ratio varies in the range of 0.6–0.8.

In Fig. 3, the average values of the reconstructed parameters in tumor regions of the three malignant patients (G1, G2 and G8) are presented, along with those estimated from microscope observation. The mean particle sizes for these three cases are 6.2 µm, 6.4 µm and 6.0 µm, respectively. The recovered values of nuclear volume fraction are 26%, 24% and 23%, respectively. The corresponding numbers for the nuclear aspect ratio are 0.59, 0.7and 0.72, respectively. The retrieved parameters are in close agreement with those estimated from the microscopic images. The normalized reconstruction error at each node of the computational domain is defined as:
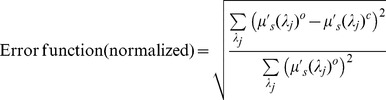
(1)Where 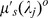
and 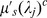
are the observed and computed reduced scattering coefficients at the wavelength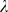, respectively [19]. For the three malignant patients, (G1, G2 and G8), average reconstruction errors drop to 17%, 20% and 18%, respectively.

In comparison with the pathological findings, as shown in Fig. 3, the errors in the three recovered parameters for patient G1 are 20%, 8% and 9%, respectively. For patient G2, the corresponding errors are 43%, 11% and 2%, respectively. Similarly for patient G8, the errors are 11%, 11% and 15%, respectively.

For each patient, we have the microscopic images from a single view, with different magnifications. They include slide views at 100X, 200X and 400X. These images contain hundreds of nuclei with slightly different sizes and elongations. For each region, we averaged out the values obtained from several nuclei (10–20), which is acceptable for estimating the nuclear parameters. We had to ignore some nuclei with very irregular and complex shapes, since it is not accurate to adapt spheroidal model for them. For DOT, we recover the parameters at 634 computational nodes. Depending on the region of interest, the values are averaged out using the data at the corresponding nodes.

In addition to the 3 malignant cases, we processed the data from 11 patients diagnosed with benign breast lesions. The scattering coefficients measured in benign cases are approximately half of those coefficients measured in malignant cases. The data from benign cases yield smaller values for the nuclear size and volume fraction. The summary of all 14 cases are presented in Fig. 4 where the average values of the three recovered parameters along with the data points are presented.

In Fig. 4a, the retrieved nuclear sizes versus nuclear volume fractions for all 14 cases are shown in four regions: malignant lesion, benign lesion, normal tissue surrounding malignant lesion and the normal tissue surrounding benign lesion. In Fig. 4b, the nuclear sizes versus aspect ratios are presented. Similarly, in Fig. 4.c, the nuclear volume fractions versus the aspect ratios are presented. The parameters, shown in Fig. 4, are averaged out over all computational nodes belonging to tumor or healthy regions. The average values of nuclear size in malignant and benign tissue are 6.1 m and 5.3 µm, respectively. The nuclear size in normal tissues surrounding the cancer lesions varies in the range of 4–5.2 µm. The average values of nuclear volume fraction in malignant and benign lesions are 23% and 9%, respectively.

In malignant and benign cases, the average values of nuclear aspect ratio are 0. 7 and 0.79, respectively, which implies more nuclear elongation in malignant cases. The three recovered parameters in malignant cases (G1, G2 and G8) show clear distinction with respect to those in benign cases.

Among the three presented graphs in Fig. 4, the malignant lesions (red data points) are adequately distinguishable from the benign lesions (blue data points) in Fig. 4a (nuclear size vs. volume fraction) and in Fig. 4c (the nuclear volume fraction vs. aspect ratio). In Fig. 4.b (nuclear size vs. aspect ratio), one benign case is retrieved in proximity of malignant cases. Another observed fact is that malignant and benign lesions are differentiable from normal tissues surrounding them in Fig. 4a and Fig. 4b. Tumor-to-tissue contrast in the retrieved nuclear size is 1.28 for malignant cases while it is 1.4 for benign cases. Tumor-to-tissue contrast in the retrieved volume fraction is 1.73 for malignant cases while it is 1.7 for benign cases. There is no significant contrast in the retrieved aspect ratio between tumor and normal tissues. However, the contrast between the retrieved aspect ratio in malignant and benign cases is 0.89.

Data reported in other reports regarding particle size, volume fraction, and aspect ratios are presented in Table 1. Different techniques are used in the relevant references to evaluate nuclear morphology parameters. In Ref. [6], automatic measurements of mean nuclear area in breast cancer cells were performed using an advanced image processing and analysis system. Similarly in Ref. [2], extensive microscopic assessments were conducted in 40 patients to recover the nuclear size and elongation in normal and Ductal carcinoma in situ (DCIS) tissues. In Ref. [14], the study included 53 malignant and 29 benign cases using fine needle aspiration cytology and microscopic evaluations. In Ref. [5], stereological studies were conducted in 42 invasive ductal breast carcinomas to estimate the nuclear count and fractions in breast tissues. In Ref. [7], a biomarker utility was employed to evaluate the nuclear area fraction which is an indicator of tumor aggressiveness. As shown in Table 1, the values reported by other researchers show similar contrast ratios in nuclear morphology parameters between the cancer and benign/healthy cases.

Our method can be considered as a non-invasive modality for breast cancer detection and classification. The tree recovered nuclear parameters (in combination as shown in Fig 4) provide an alternative, and sometimes a more reliable, tool for this purpose. For instance, for patient with ID# G8, the cancer (carcinoma *in situ*) was not revealed by mammography whereas it was categorized as a malignant case using the nuclear morphology images obtained from our technique.

The results obtained from a few number of patients are not statistically enough to draw general conclusions. While we have not been able to continue to collect data from a much larger patient set due to the lack of funding for the last four years in this area, the primary purpose of the current study is to present an improved analysis method that shows the potential for improved detection of breast cancer. We hope the funding situation will change in the coming years which would then allow us to conduct a larger clinical trial for statistically significant conclusions.

### Conclusions

In an ongoing effort to develop a non-invasive and efficient tool for breast cancer detection and classification in vivo, we herein report the results obtained from our T-matrix based inverse algorithm, based on *in-vivo* measurements at the breast skin. Our results show that nuclei of cancer cells are larger, more crowded and more elongated and these features of cancer cells can be determined non-invasively using DOT. Our non-invasive imaging results are in agreement with the microscopic images obtained from direct tissue sampling. This study highlights the applicability of a light-scattering-based modality for breast cancer determination.
